# Supplementary material for: Xiphoid nucleus of the midline thalamus controls cold-induced food seeking
Source: Nature. 2023 Aug 16;621(7977):138–45. doi: 10.1038/s41586-023-06430-9 (PMC10482681; doi:10.1038/s41586-023-06430-9)
Supplement: Supplementary file 2 — Reporting Summary [file 41586_2023_6430_MOESM2_ESM.pdf]

nature portfolio

Corresponding author(s): Li Ye

Last updated by author(s): 03/02/2023 04 / 27 / 2023

Reporting Summary

Nature Portfolio wishes to improve the reproducibility of the work that we publish. This form provides structure for consistency and transparency in reporting. For further information on Nature Portfolio policies, see our Editorial Policies and the Editorial Policy Checklist.

Statistics

For all statistical analyses, confirm that the following items are present in the figure legend, table legend, main text, or Methods section.

n/a	Confirmed	
☐	☒	The exact sample size (*n*) for each experimental group/condition, given as a discrete number and unit of measurement
☐	☒	A statement on whether measurements were taken from distinct samples or whether the same sample was measured repeatedly
☐	☒	The statistical test(s) used AND whether they are one- or two-sided<br>*Only common tests should be described solely by name; describe more complex techniques in the Methods section.*
☐	☒	A description of all covariates tested
☐	☒	A description of any assumptions or corrections, such as tests of normality and adjustment for multiple comparisons
☐	☒	A full description of the statistical parameters including central tendency (e.g. means) or other basic estimates (e.g. regression coefficient) AND variation (e.g. standard deviation) or associated estimates of uncertainty (e.g. confidence intervals)
☐	☒	For null hypothesis testing, the test statistic (e.g. *F*, *t*, *r*) with confidence intervals, effect sizes, degrees of freedom and *P* value noted<br>*Give P values as exact values whenever suitable.*
☒	☐	For Bayesian analysis, information on the choice of priors and Markov chain Monte Carlo settings
☒	☐	For hierarchical and complex designs, identification of the appropriate level for tests and full reporting of outcomes
☒	☐	Estimates of effect sizes (e.g. Cohen's *d*, Pearson's *r*), indicating how they were calculated

Our web collection on statistics for biologists contains articles on many of the points above.

Software and code

Policy information about availability of computer code

Data collection	Imaging data for histology was collected using olympur FV31S-SW (version 2.5.1.228. powered by H-PF Version 2.123.2.139).Fiberphotometry data was collected using custom MATLAB (version 2018b) code. Metaboic data was collected with CalR
Data analysis	Statistical calculations were performed using GraphPad Prism 9 (GraphPad Software, Inc., La Jolla, CA) and Microsoft Excel 2020. MATLAB (version 2018b) code were used for the analysis of fiberphotometry and HMM data. Behavior data was scored manually using QuickTime 10.4. Imaging data was analyzed using ImageJ(FIJI, 2.3.0).IHC images were prepared for publication using image J (FIJI, 2.3.0).

For manuscripts utilizing custom algorithms or software that are central to the research but not yet described in published literature, software must be made available to editors and reviewers. We strongly encourage code deposition in a community repository (e.g. GitHub). See the Nature Portfolio guidelines for submitting code & software for further information.

Data

Policy information about availability of data

All manuscripts must include a data availability statement. This statement should provide the following information, where applicable:

- Accession codes, unique identifiers, or web links for publicly available datasets
- A description of any restrictions on data availability
- For clinical datasets or third party data, please ensure that the statement adheres to our policy

All numerical data is included in the supplementary information. All other data is too large to deposit in a public repository and is available from corresponding author. Source data is provided with the manuscript. Code used in this study can be accessed at Zenodo https:/ / zenodo.org / record / 7869467#.ZEnDEezMLmE. Code D0I: 10.5281 / zenodo.7869467

Human research participants

Policy information about studies involving human research participants and Sex and Gender in Research.

Reporting on sex and gender	*Not applicable*
Population characteristics	*Not applicable*
Recruitment	*Not applicable*
Ethics oversight	*Not applicable*

Note that full information on the approval of the study protocol must also be provided in the manuscript.

Field-specific reporting

Please select the one below that is the best fit for your research. If you are not sure, read the appropriate sections before making your selection.

☒ Life sciences ☐ Behavioural & social sciences ☐ Ecological, evolutionary & environmental sciences

For a reference copy of the document with all sections, see nature.com/documents/nr-reporting-summary-flat.pdf

Life sciences study design

All studies must disclose on these points even when the disclosure is negative.

Sample size	No analysis were performed in advance to predetermine the sample size. Sample size were based on previously published papers in the literature:<br><br>1) Hrvatin, S., Sun, S., Wilcox, O.F. et al. Neurons that regulate mouse torpor. Nature 583, 115–121 (2020). https://doi.org/10.1038/s41586-020-2387-5<br><br>2) Salay, L.D., Ishiko, N. & Huberman, A.D. A midline thalamic circuit determines reactions to visual threat. Nature 557, 183–189 (2018). https://doi.org/10.1038/s41586-018-0078-2
Data exclusions	Data from 3 mice were excluded from figure 2J based on post-hoc analysis of injection site. Data from 2 mice were excluded from Figure 4 C and E based on post-hoc analysis of injection and implantation site.
Replication	All the experiments were repeated more than once. All attempts at replication were successful.
Randomization	Animals of same age, sex and weight were randomly assigned into treatment or control group.
Blinding	Cell counting, fiber photometry data and HMM analysis was performed by person blinded to experimental condition. For all other experiments experimenter was not blinded because the researcher need to know the site of implantation and injection to exclude failed surgeries.

Reporting for specific materials, systems and methods

We require information from authors about some types of materials, experimental systems and methods used in many studies. Here, indicate whether each material, system or method listed is relevant to your study. If you are not sure if a list item applies to your research, read the appropriate section before selecting a response.

Materials & experimental systems

n/a	Involved in the study
☐	☒ Antibodies
☒	☐ Eukaryotic cell lines
☒	☐ Palaeontology and archaeology
☐	☒ Animals and other organisms
☒	☐ Clinical data
☒	☐ Dual use research of concern

Methods

n/a	Involved in the study
☒	☐ ChIP-seq
☒	☐ Flow cytometry
☒	☐ MRI-based neuroimaging

Antibodies

Antibodies used

cFOS antibody (Cell signaling catalog number 2250) (diluted 1:400) , Anti-Rabbit-488(Jackson Immuno Research 711-546-152, dilution of 1:400)

Validation

All antibodies were sourced from well established companies and are thoroughly validated by manufacturer and is widely used in neuroscience literature.
cFOS antibody (Cell signaling 2250)
https://www.cellsignal.com/products/primary-antibodies/c-fos-9f6-rabbit-mab/2250

Animals and other research organisms

Policy information about studies involving animals; ARRIVE guidelines recommended for reporting animal research, and Sex and Gender in Research

Laboratory animals

Mice were housed with standard 12 hour light/dark cycle at 23C. The lights were on from 6 am to 6 pm. All experiments were performed on wild type C57BL6/J mice ordered from the Scripps Research Department of Animal Resources rodent breeding colony. Experiments were performed on mice between the age of 2 to 6 months.

Wild animals

No wild animals were used.

Reporting on sex

The findings reported here apply to both sexes. The initial screening was performed in both sexes. cFOS data for both sexes are reported separately in Fig. 2B and Fig. S4A

Field-collected samples

No field-collected samples were used.

Ethics oversight

Animal experiments were performed either at Scripps Research Institute, La Jolla or Beth Israel Deaconess Medical Center (BIDMC). Experiments were approved by the Scripps Research Institute's or BIDMC's Institutional Animal Care and Use Committee (IACUC), respectively. All experiments were in accordance with the guidelines from the NIH.

Note that full information on the approval of the study protocol must also be provided in the manuscript.
